# Oncofetal HMGA2 attenuates genotoxic damage induced by topoisomerase II target compounds through the regulation of local DNA topology

**DOI:** 10.1002/1878-0261.12541

**Published:** 2019-08-31

**Authors:** Syed Moiz Ahmed, Peter Dröge

**Affiliations:** ^1^ School of Biological Sciences Nanyang Technological University Singapore Singapore

**Keywords:** chemotherapy, DNA topology, HMGA2, replication stress, TOP2

## Abstract

Rapidly dividing cells maintain chromatin supercoiling homeostasis via two specialized classes of enzymes, DNA topoisomerase type 1 and 2 (TOP1/2). Several important anticancer drugs perturb this homeostasis by targeting TOP1/2, thereby generating genotoxic DNA damage. Our recent studies indicated that the oncofetal chromatin structuring high‐mobility group AT‐hook 2 (HMGA2) protein plays an important role as a DNA replication fork chaperone in coping with DNA topological ramifications that occur during replication stress, both genomewide and at fragile sites such as subtelomeres. Intriguingly, a recent large‐scale clinical study identified *HMGA2* expression as a sole predicting marker for relapse and poor clinical outcomes in 350 acute myeloid leukemia (AML) patients receiving combinatorial treatments that targeted TOP2 and replicative DNA synthesis. Here, we demonstrate that HMGA2 significantly enhanced the DNA supercoil relaxation activity of the drug target TOP2A and that this activator function is mechanistically linked to HMGA2's known ability to constrain DNA supercoils within highly compacted ternary complexes. Furthermore, we show that HMGA2 significantly reduced genotoxic DNA damage in each tested cancer cell model during treatment with the TOP2A poison etoposide or the catalytic TOP2A inhibitor merbarone. Taken together with the recent clinical data obtained with AML patients targeted with TOP2 poisons, our study suggests a novel mechanism of cancer chemoresistance toward combination therapies administering TOP2 poisons or inhibitors. We therefore strongly argue for the future implementation of trials of HMGA2 expression profiling to stratify patients before finalizing clinical treatment regimes.

**Abbreviations**
AML: acute myeloid leukemia
Dox: doxycycline
DSB: double‐strand break
Etop: etoposide
HMGA2: high‐mobility group AT‐hook 2
Merb: merbarone
scDNA: supercoiled DNA
TOP1/2: topoisomerase 1/2
TOP2cc: TOP2‐DNA cleavage complexes

## 1 Introduction

The fine‐tuned regulation of DNA/chromatin topology is essential for the maintenance of cellular functions and genome stability. This applies especially to fast proliferating cancer cells which face severe DNA topological challenges during genome replication and other DNA transactions (Droge, 1994; Keszthelyi *et al.*, 2016; Kotsantis, *et al.*, 2018; Wang, 2002). DNA topoisomerase type 1 and 2 (TOP1/2) introduce controlled, transient breaks into chromosomal DNA, thereby resolving DNA/chromatin topological ramifications and promoting DNA transactions such as the progression of replication forks and RNA polymerases (Bermejo *et al.*, 2007; Delgado *et al.*, 2018; Wang, 1985; Zechiedrich and Osheroff, 1990).

Given these crucial cellular functions, a diverse group of anticancer drugs that target human TOP2 are often administered as the first‐line therapy to treat primary as well as metastatic tumors (Hande, 1998a; Hevener *et al.*, 2018; Pommier, 2013; Pommier *et al.*, 2010). Etoposide (Etop) or VP‐16 and doxorubicin are the most prescribed TOP2 inhibitors applied alone or in multidrug treatment regimens. Etop is widely used in the treatment of testicular, small cell lung cancers, and lymphomas (Hande, 1998b; Hevener *et al.*, 2018), whereas doxorubicin is often administered to treat acute myeloid leukemia (AML) and several other carcinomas (Burden and Osheroff, 1998; Meredith and Dass, 2016; Tacar *et al.*, 2013).

Most clinically relevant TOP2 inhibitors lead to the formation of covalent TOP2‐DNA cleavage complexes (TOP2cc) by preventing religation of the double‐strand break (DSB) in otherwise transiently cleaved DNA molecules (Chikamori *et al.*, 2010; Hu *et al.*, 2018; Osheroff, 1989). These inhibitors are collectively called TOP2 ‘poisons’ and cause genomewide DNA strand breaks, for example, during DNA transactions when replisomes or transcription assemblies collide with TOP2cc (Baranello *et al.*, 2014; Pommier *et al.*, 2010; Tammaro *et al.*, 2013; Yan *et al.*, 2016; Yu *et al.*, 2017). TOP2 catalytic inhibitors, for example, merbarone (Merb), do not trap the enzyme in covalent complexes with DNA (TOP2cc), but they also block DNA supercoil relaxation. This class of inhibitors is under clinical evaluation and can induce DNA damage. However, their genotoxic mechanisms of action remain to be elucidated, with inducing DNA replication stress and replication fork collapse as strong candidates (Fortune and Osheroff, 1998; Glover *et al.*, 1987; Larsen *et al.*, 2003; Nitiss, 2009; Pastor *et al.*, 2012; Wang and Eastmond, 2002; Wang *et al.*, 2007).

Although TOP2‐targeting anticancer drugs have successfully been administered in the clinic, the treatment outcome per individual patient varies greatly. Hence, a better understanding of cancer cell‐autonomous factors which determine their treatment efficacies could have clinical impact. In this context, the high‐mobility group AT‐hook 2 (HMGA2) protein is known as a nonhistone architectural chromatin factor which has been implicated in carcinogenesis and in particular metastasis (Gao *et al.*, 2017; Morishita *et al.*, 2013; Pallante *et al.*, 2015; Young and Narita, 2007). The protein is not detectable in most adult somatic cells, but *HMGA2* is aberrantly expressed during malignant cell transformation, particularly in mesenchymal tumors (Dreux *et al.*, 2010). Its elevated expression in breast cancer (Wu *et al.*, 2016), lung cancer (Sarhadi *et al.*, 2006), colorectal cancer (Wang *et al.*, 2011), oral carcinomas (Miyazawa *et al.*, 2004), and several other malignancies has often been associated with poor clinical treatment outcomes (Wu and Wei, 2013).

High‐mobility group AT‐hook 2 possesses three independent DNA binding domains, which preferentially recognize AT‐rich nucleotide sequences in double‐stranded DNA (Pfannkuche *et al.*, 2009; Reeves and Nissen, 1990). Besides important regulatory roles in gene expression, in particular during embryonic/fetal development and tumorigenesis/metastasis in the adult organism (Droge and Davey, 2008; Fusco and Fedele, 2007; Pfannkuche *et al.*, 2009; Sgarra *et al.*, 2018), HMGA2 has also been implicated in DNA base excision repair (Summer *et al.*, 2009) and DNA damage repair signaling pathways (Hombach‐Klonisch *et al.*, 2019; Natarajan *et al.*, 2013; Palmieri *et al.*, 2011), hinting at important functions for HMGA2 in genome stability following genotoxic stress conditions. In this context, fast proliferation rates of cancer cells enhance DNA replication stress (Gaillard *et al.*, 2015), and we have recently demonstrated that HMGA2 broadly protects hydroxyurea (HU)‐induced stalled replication forks from collapse into genotoxic DSBs in human cancer and stem cells, thus implying that HMGA2 is also involved upstream of DNA repair processes as a first line of defense to prevent genome instability (Ahmed *et al.*, 2019; Yu *et al.*, 2014).

High‐mobility group AT‐hook 2, by means of its three AT‐hook DNA binding domains, forms unique complexes with supercoiled DNA (scDNA), thereby curbing excess topological stress via constrainment of scDNA (Ahmed *et al.*, 2019; Peter *et al.*, 2016; Zhao *et al.*, 2017). Intriguingly, depending on the relative HMGA2 expression levels in various cancer cell models, HMGA2 either triggered or attenuated the genotoxic action of the clinically important TOP1 poison irinotecan/SN38, specifically at heterochromatic subtelomeres (Ahmed *et al.*, 2019). The sensitizing effect of low‐to‐moderate HMGA2 expression to irinotecan treatment was also demonstrated in patient‐derived xenograft models of human colon cancer, and we mechanistically ascribed these outcomes to a potentiating drug effect in ternary scDNA‐TOP1‐HMGA2 complexes. Collectively, these data led to a new model in which HMGA2 plays an important role in cancer genome stability at topologically challenged chromatin regions, such as replication forks and subtelomeres (Ahmed *et al.*, 2019; Zhao *et al.*, 2017).

Here, we show that the expression of HMGA2 confers broad protection to cancer cells treated with the clinically important TOP2‐targeting drugs Etop and Merb, leading to a significant reduction in genotoxic DSBs during the course of drug treatment. We propose that the DNA topological stress generated through the drug‐induced catalytic inhibition and/or the trapping of TOP2 with DNA in chromatin is counteracted by HMGA2 via a combination of TOP2 catalytic activation and DNA supercoil constrainment. Importantly, our proposed model provides a plausible mechanistic explanation for the results of a recently published clinical study (Marquis *et al.*, 2018) with more than 350 AML patients who were treated with TOP2 poisons in combination with the DNA synthesis inhibitor cytarabine (Ara‐C) as first‐line combination therapy. The clinical data revealed that *HMGA2* expression in leukemic cells *in vivo* is an independent negative predictor of disease relapse and patient survival (Marquis *et al.*, 2018). Taken together with our previous HMGA2 studies investigating TOP1 poisons and DNA synthesis inhibitors (Ahmed *et al.*, 2019; Yu *et al.*, 2014), our current study strongly enforces the clinical importance of HMGA2 as a prognostic therapeutic marker in the clinic and as an important drug target.

## 2 Materials and methods

### 2.1 Cell culture

All cancer cell line model systems were cultured according to ATCC recommendations. Recombinant HT1080 C1/C2 cells expressing doxycycline (Dox) hyclate (Sigma, Singapore) inducible shHMGA2 were generated as described previously (Yu *et al.*, 2014), and HMGA2 knockdown was achieved with Dox treatment once every day for 4 days. Human HMGA2‐expressing clones A549 (1.3/1.5/1.6) and HeLa (P2/P8/P19) have been described previously (Summer *et al.*, 2009). H1299 HMGA2 KO cells were generated by CRISPR‐Cas9 and have also been described previously (Ahmed *et al.*, 2019).

### 2.2 Western blotting

Protein lysates were prepared as detailed previously (Ahmed *et al.*, 2019). Briefly, cells were resuspended in RIPA buffer containing Protease Inhibitor cocktail (Roche, Basel, Switzerland) on ice. The mixture was then centrifuged at 15 000 r.p.m. for 20 min at 4 °C. The supernatant was collected, and following protein concentration determination, lysates were separated by SDS/PAGE, transferred onto 0.2‐µm PVDF membranes (Bio‐Rad, Hercules, CA, USA) and blocked in superblock blocking buffer (Thermo Fisher Scientific, Waltham, MA, USA). Membranes were incubated with primary antibodies [α‐HMGA2 (CST 5269; 1 : 1000; RRID:AB_10694917), α‐TOP2A (TopoGEN TG2011‐1; 1 : 2000; RRID:AB_1934304), α‐TOP2B (Ab109524; 1 : 2000; RRID:AB_10859793), α‐β‐actin (Sigma A2228; 1 : 5000; RRID:AB_476697)] overnight at 4 °C, followed by incubation with secondary antibodies [Polyclonal goat anti‐mouse (Dako, P0447, Carpinteria, CA, USA; RRID:AB_2617137) and polyclonal goat anti‐rabbit (Dako, P0448; RRID:AB_2617138)] at room temperature for 1 h. Immunoreactivity was developed by chemiluminescent HRP substrate (Millipore, Singapore) in a luminescence imager (LAS4000; Fujifilm, Pittsburgh, PA, USA).

### 2.3 Pulsed‐field gel electrophoresis (PFGE) and Southern blotting

Our pulsed‐field gel electrophoresis (PFGE) assay conditions have been described previously (Ahmed *et al.*, 2019). Briefly, 0.4 × 10^6^ cells were seeded in a six‐well tissue culture plate and treated with indicated doses of Etop (Sigma) for 24 h and Merb (Santa Cruz Biotechnology, Dallas, TX, USA) for 48 h, respectively. 48 h drug treatment involved media replacement with fresh drug added every 24 h. DMSO‐treated cells were used as control. Harvested cells were embedded in 2% low melting agarose (Sigma) plugs followed by incubation in lysis buffer (0.2% sodium deoxycholate, 1% sodium lauroyl sarcosine, 100 mm EDTA, and 1 mg·mL^−1^ proteinase K) at 50 °C for 24 h. Plugs were washed four times in TE buffer for 1 h each and electrophoresed through 1% megabase agarose (Bio‐Rad) on CHEF DR II (Bio‐Rad). Lambda PFG ladder (NEB, Ipswich, MA, USA) that ranges from 48.5 to 1018 kb was used as a marker. Subsequently, PFGE gels were stained with ethidium bromide, and quantification was performed using imagej software, as described in detail in Figure legends. Following PFGE, telomeric DNA was detected by Southern blotting using TeloTAGGG Telomere Length Assay kit (Roche, 12209136001) as per the manufacturer's protocol and previously detailed in (Ahmed *et al.*, 2019).

### 2.4 *In vitro* Etop‐induced DNA cleavage assay

Indicated amounts of Etop (Sigma) diluted in DMSO were incubated with 100 ng of supercoiled Renilla reporter plasmid (Peter *et al.*, 2016) in a buffer containing 10 mm Tris/HCl, pH 7.9, 50 mm KCl, 50 mm NaCl, 5 mm MgCl_2_, 0.1 mm EDTA, 1 mm ATP, 15 µg·mL^−1^ BSA. The DNA supercoil relaxation reactions were initiated by adding 4.5 U of human recombinant TOP2A (Affymetrix, Santa Clara, CA, USA) to each sample. Reactions were stopped after 30 min at 37 °C with 0.3% (w/v) SDS followed by proteinase K (Thermo Scientific) digestion for 20 min at 37 °C. Samples were analyzed on 0.8% agarose gels and visualized with ethidium bromide staining under UV.

### 2.5 *In vitro* HMGA2 DNA relaxation assay

One hundred nanogram of supercoiled plasmid DNA was incubated with different amounts of either purified wild‐type HMGA2 or 2,3 AT‐hook mutant HMGA2 protein and 0.12 U of human TOP2A (Affymetrix) for 30 min at 37 °C in a buffer containing 10 mm Tris/HCl, pH 7.9, 50 mm KCl, 50 mm NaCl, 5 mm MgCl_2_, 0.1 mm EDTA, 1 mm ATP, 15 µg·mL^−1^ BSA. In test runs, we had first established that 0.12 U of human TOP2A achieved partial DNA relaxation in the absence of HMGA2, hence allowing us to investigate catalytic activation functions. Reactions were stopped with 0.3% (w/v) SDS followed by proteinase K digestion. Samples were electrophoresed overnight on a 0.8% agarose gel and visualized under UV by staining with ethidium bromide.

### 2.6 Complementation assay

4 × 10^5^ HeLa cells were seeded in a six‐well plate. DNA transfection was done using lipofectamine 2000 (Invitrogen, 11668‐019, Carlsbad, CA, USA) as per the manufacturer's instructions. pEF1/*Myc*‐A‐*hmga2*‐FLAG was used for expression of wild‐type HMGA2, whereas pEF1/*Myc*‐A‐*hmga2*‐hook2/3Mutant‐FLAG was used to express 2,3hook HMGA2 mutant, with pEF1/*Myc*‐A used as mock control. Thirty‐six hours post‐transfection, cells were exposed to Etop for 24 h followed by analysis of DSBs by PFGE as described in section 2.3. All transfected plasmids have been described previously (Yu *et al.*, 2014).

### 2.7 Cell survival assay

Cells were seeded as triplicates in a 96‐well black/clear bottom plate for each condition and 12 h later treated with indicated doses of Etop/Merb for 48 h. The drug treatment involved media replacement with fresh drug added every 24 h. Cell viability was determined using the CCK‐8 assay (Enzo Life Sciences, ALX‐850‐039‐0100, Singapore) as per the manufacturer's instructions. Briefly, 10 µL of CCK‐8 solution is directly added to each well of the plate and incubated for 2 h at 37 °C. Absorbance was measured at 450 nm using microplate reader (TECAN Infinite M200 Pro, Tecan Trading AG, Mannedorf, Switzerland). The mean absorbance of untreated triplicates was used for normalization.

### 2.8 Statistical analysis

All quantitative PFGE data are represented as mean ± standard deviation (SD), calculated from at least three independent experiments. Statistical significance is calculated using unpaired two‐tailed *t*‐test. *P* < 0.05 was considered statistically significant with *P < 0.05, **P < 0.01, ***P < 0.001. Data were plotted using graphpad prism software (RRID:SCR_002798GraphPad Software, San Diego, CA, USA).

## 3 Results

### 3.1 HMGA2 protects cancer cells against Etop‐induced replication stress

Both TOP1 and TOP2 play key roles in the relaxation of transient DNA supercoiling that occurs during replication and other DNA transactions, such as transcription (Bermejo *et al.*, 2007; Droge, 1994; McClendon *et al.*, 2005; Pommier *et al.*, 2016; Wang, 2002). Our previous studies have shown that HMGA2 functions as a replication fork chaperone when replication is challenged by the DNA synthesis inhibitor HU (Yu *et al.*, 2014). However, HMGA2 differentially affects the genotoxic efficacy of the clinically relevant TOP1 poison irinotecan/SN38, and we mechanistically ascribed these different phenotypic outcomes of drug treatment to ternary complex formation between HMGA2, TOP1, and scDNA substrates (Ahmed *et al.*, 2019). Here, by employing our four previously established cancer cell models, we first investigated whether HMGA2 affects DNA damage caused by the clinically relevant TOP2 poison Etop.

**Figure 1:**
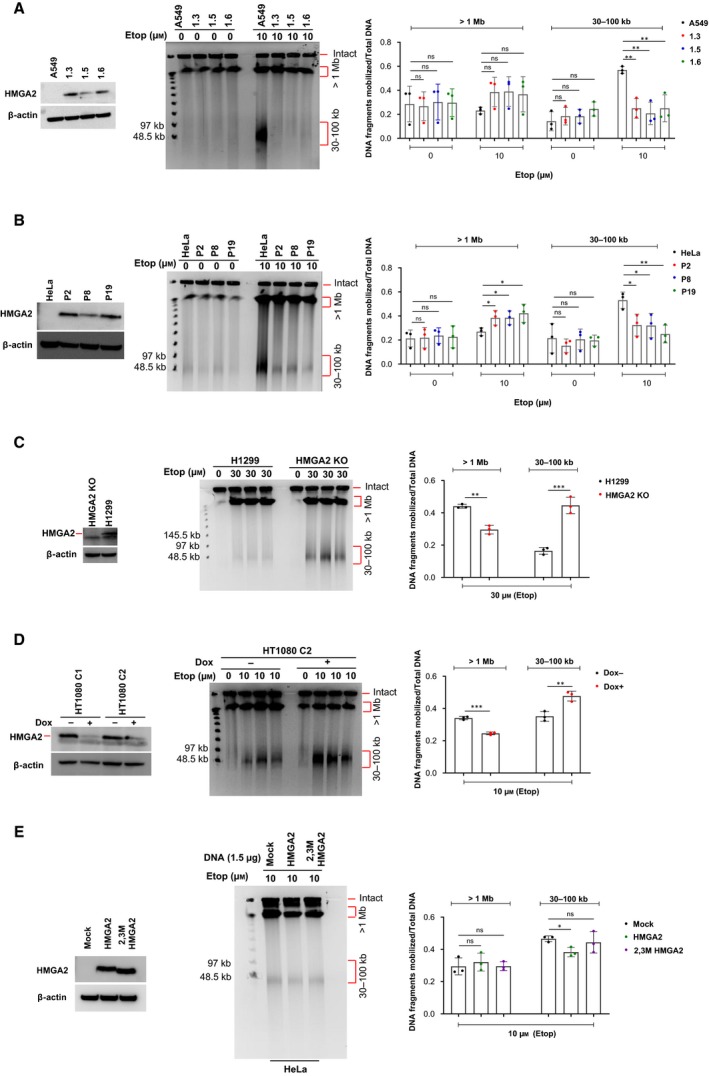
HMGA2 protects against DNA damage induced by Etop. (A) A549 cells (parental and three recombinant human HMGA2‐expressing clonal cell lines; left panel) were treated with 10 μm Etop for 24 h, and their DNA was analyzed for DSB formation by PFGE. DMSO‐treated cells served as controls (center panel). Quantification of Etop‐induced DNA fragments (> 1 Mb and 30–100 kb fractions; right panel) after normalization with total DNA (*n* = 3 independent experiments). (B) HeLa cells (parental and three recombinant human HMGA2‐expressing clonal cell lines; left panel) were treated as described for A549 cells in (A). DMSO‐treated cells were used as controls (center panel). Quantification of Etop‐induced DNA fragments (> 1 Mb and 30–100 kb fractions; right panel) after normalization with total DNA (*n* = 3 independent experiments). Also, see Fig. S1A for an independent Etop titration analysis in HeLa cells. (C) H1299 cells (HMGA2 KO and parental; left panel) were treated with 30 μm Etop for 24 h and their DNA was analyzed for DSB formation by PFGE. DMSO‐treated cells were used as controls (center panel). Quantification of Etop‐induced DNA fragments (> 1 Mb and 30–100 kb fractions; right panel) after normalization with total DNA (*n* = 3 independent experiments). Also, see Fig. S1B for an independent dose titration analysis. (D) HT1080 C1/2 cells with Dox‐regulated HMGA2 expression (left panel) were treated with 10 μm Etop for 24 h, and their DNA was analyzed by PFGE. DMSO‐treated cells were used as controls (center panel). Quantification of Etop‐induced DNA fragments (> 1 Mb and 30–100 kb fractions) obtained with C2 cells (right panel) after normalization with total DNA (*n* = 3 independent experiments). Fig. S1C shows the results obtained with HT1080 C1 cells. (E) HeLa cells transiently transfected with expression vectors for wild‐type HMGA2, 2,3‐hook mutant HMGA2 and mock vector as control (HMGA2 Western blot in left panel) were treated with 10 μm Etop for 24 h and their DNA was analyzed by PFGE (center panel). Quantification of DNA fragments (> 1 Mb and 30–100 kb fractions; right panel) after normalization with total DNA (*n* = 3 independent experiments). (A–E) See section 2.8 for statistical analysis.

**Figure 2:**
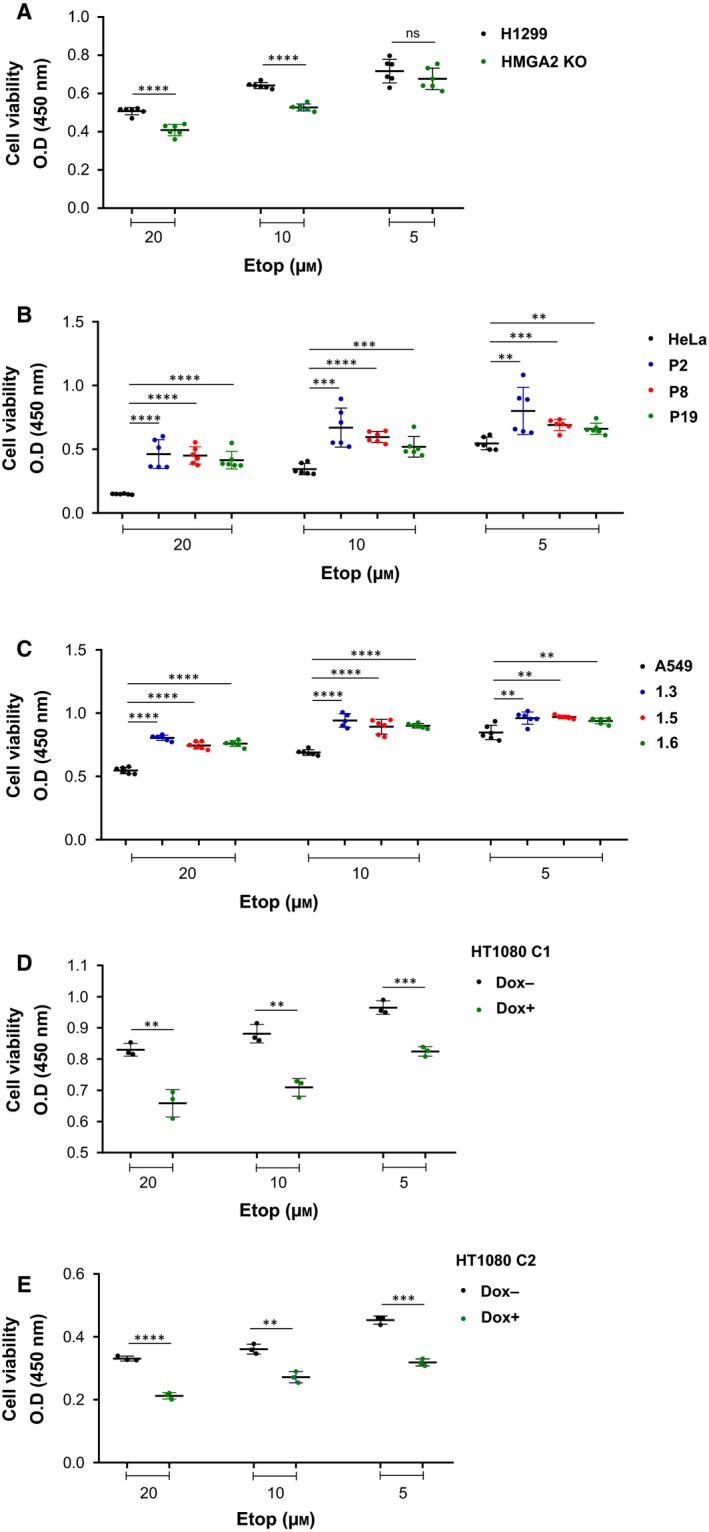
Effects of HMGA2 expression on cell viability during Etop treatment. (A) Cell viability (CCK8) assay with H1299 (parental and HMGA2 knockout) cells after treatment with three different doses of Etop for 48 h without recovery incubation (*n* = 2 independent experiments with 3 technical replicates for each experiment). Fresh drug was added every 24 h during media replacement. (B) Cell viability (CCK8) assay with HeLa (parental cells and three HMGA2 expressing cell clones) cells after treatment with three different doses of Etop for 48 h (*n* = 2 independent experiments with three technical replicates for each experiment). (C) Cell viability (CCK8) assay with A549 (parental cells and three HMGA2 expressing cell clones) cells after treatment with three different doses of Etop for 48 h (*n* = 2 independent experiments with three technical replicates for each experiment). (D) Cell viability (CCK8) assay with HT1080 C1 (Dox−/Dox+) cells after treatment with three different doses of Etop for 48 h. (*n* = 1 independent experiment with 3 technical replicates for each experiment). (E) Cell viability (CCK8) assay with HT1080 C2 (Dox−/Dox+) cells after treatment with three different doses of Etop for 48 h. (*n* = 1 independent experiment with three technical replicates for each experiment). (A–E) Mean of untreated controls used for normalization. Unpaired two‐tailed *t*‐tests. Error bars, SD. **P < 0.01, ***P < 0.001, ****P < 0.0001, ns: not significant.

The induction of DNA DSBs after 24 h Etop exposure without recovery time was evaluated by PFGE, which revealed two distinct genome fragment fractions (megabase‐sized and 30‐ to 100‐kb‐sized DNA fragments; Fig. 1A–D and Fig. S1A–C). We found that the expression of HMGA2 always reduced the occurrence of DSBs that generated the drug‐induced 30–100 kb DNA fragments across all four cancer cell model systems. These models exhibit differential HMGA2 expression levels due to either *HMGA2* knockout (H1299 cells), HMGA2 knockdown (HT1080 cells), or HMGA2 overexpression (HeLa and A549 cells) (Ahmed *et al.*, 2019). Furthermore, human HMGA2 expressed from a transiently transfected vector in parental HeLa cells that do not express detectable levels of endogenous HMGA2 confirmed a specific protective function of HMGA2 against Etop‐induced DNA breaks (Fig. 1E). This complementation assay also included a variant HMGA2 protein in which two (i.e., AT‐hooks 2 and 3) of the three individual DNA binding domains of HMGA2 carried amino acid substitutions (2,3M HMGA2) that reduced their binding affinities to DNA (Yu *et al.*, 2014). This variant HMGA2 failed to complement the lack of wild‐type HMGA2 in HeLa cells. These results highlighted that DNA binding via HMGA2's AT‐hooks is critical for its protective function against Etop (Fig. 1E). To validate our results, we utilized cell viability assays and found that expression of HMGA2 correlated with increased cell survival during Etop treatment in all four cancer cell models (Fig. 2A–E).

### 3.2 HMGA2 does not affect TOP2 expression

**Figure 3:**
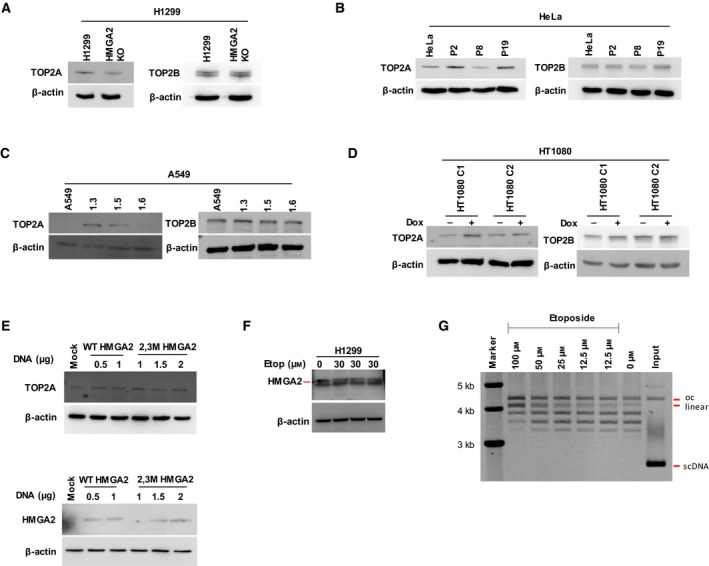
Sensitivity to TOP2 poison/inhibitor does not correlate with TOP2A/TOP2B expression. (A) Western blot of human TOP2A (left panel) and TOP2B (right panel) for H1299 cells (parental and HMGA2 KO cells). β‐Actin served as internal control for all Western blots shown in this Figure. (B) Western blot of human TOP2A (left panel) and TOP2B (right panel) for HeLa cells (parental cells and three recombinant HMGA2‐expressing cell lines). (C) Western blot of human TOP2A (left panel) and TOP2B (right panel) for A549 cells (parental cells and three recombinant HMGA2‐expressing cell lines). (D) Western blot of human TOP2A (left panel) and TOP2B (right panel) for HT1080 C1/C2 cells (Dox−/+ treated). Note that no strict correlation existed between HMGA2 and TOP2A expression levels; while TOP2A levels were slightly higher in HMGA2‐expressing H1299, HeLa and A549 cells, the opposite was observed in HT1080 cells after Dox treatment. (E) Western blot of human TOP2A (top panel) after complementation with varying amounts of wild‐type and 2,3M HMGA2 expression vector (bottom panel) in HeLa cells. Mock vector was used as transfected control DNA. (F) Western blot of HMGA2 after 30 µm Etop treatment (*n* = 3 independent experiments) for 24 h. Note that the bottom band results from a nonspecific signal. (G) Representative Etop dose titration for trapping of TOP2A‐DNA complexes generating linearized DNA *in vitro*. The positions of open circular (oc) and linearized plasmid DNA are indicated. The unlabeled DNA bands are topoisomers that result from supercoil relaxation by TOP2A. The position of the input scDNA is also indicated (*n* = 3 independent experiments). DNA was subjected to electrophoresis in 0.8% agarose gel followed by ethidium bromide staining.

With higher TOP levels being potentially accountable for enhanced drug sensitivity (Burgess *et al.*, 2008; MacGrogan *et al.*, 2003; Sevim *et al.*, 2011), we determined whether differential expression of TOP2A and TOP2B, the two major isoforms of human TOP2, might be regulated by HMGA2 and thus could be responsible for the Etop treatment outcome. Western blot analysis revealed that the observed protective effect was not due to changes in TOP2A/B expression levels that consistently correlated with HMGA2 levels (Fig. 3A––D). To corroborate this conclusion, we performed transient transfection experiments and complemented HeLa cells with wild‐type or the 2,3M HMGA2 variant and observed no corresponding changes in TOP2A expression compared to mock‐transfected controls (Fig. 3E). Furthermore, Etop treatment of H1299 cells did not alter the expression level of HMGA2, hence ruling out drug‐induced HMGA2 expression changes as a cause for the protective phenotypes (Fig. 3F).

Etop traps and stabilizes covalent TOP2cc, which can be converted into DSBs during replication runoff events (McClendon and Osheroff, 2007; Tammaro *et al.*, 2013; Yan *et al.*, 2016). We confirmed that our batch of the Etop drug is active in inducing TOP2cc formation by using supercoiled plasmid DNA and recombinant human TOP2A in *in vitro* DNA supercoil relaxation assays. Titration of various concentrations of the drug revealed that plasmid DNA linearization due to the formation of TOP2cc is induced at Etop concentrations used in our cell‐based assays (i.e., 10–30 µm; Fig. 3G). Collectively, these data suggest that HMGA2 attenuates DSB formation and cell death triggered by Etop and that DNA binding of HMGA2 is critical for this function.

### 3.3 HMGA2 counteracts topological stress at human subtelomeres and catalytically activates TOP2A

We next explored the possibility of a more direct role for HMGA2 in regulating topological stress when TOP2 is inhibited rather than ‘poisoned’. We utilized Merb, a catalytic inhibitor of TOP2 that does not stabilize cleavage complexes (TOP2cc) that would result in replication (transcription) runoff at lesions to generate DSBs, but negatively affects the supercoil relaxation activity of TOP2 (Burden and Osheroff, 1998; Chen and Beck, 1995; Tripathi *et al.*, 2019; Zhang *et al.*, 2017). It has been argued that Merb causes DNA damage by fork stalling and collapse as a result of the buildup of DNA superhelical tension in the unreplicated parental DNA (Fortune and Osheroff, 1998; Wang *et al.*, 2007).

**Figure 4:**
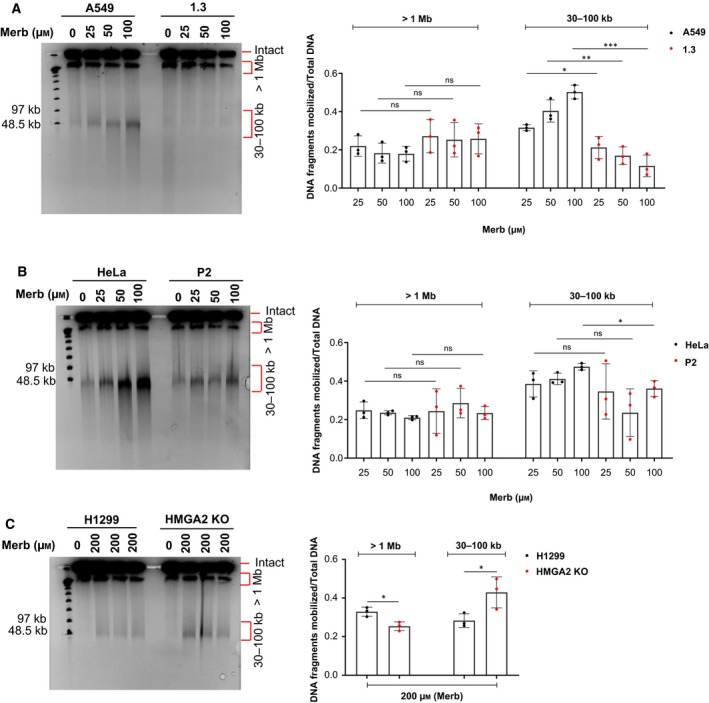
HMGA2 protects against catalytic TOP2 inactivation *ex vivo*. (A) A549 cells (parental and HMGA2 expressing cell line 1.3) were treated with increasing concentrations of Merb for 48 h and their DNA was analyzed by PFGE. DMSO‐treated cells were used as controls (left panel). Fresh drug was added every 24 h during media replacement. Quantification of Merb‐induced DNA fragments (> 1 Mb and 30–100 kb fractions; right panel) after normalization with total DNA (*n* = 3 independent experiments). (B) HeLa cells (parental and HMGA2 expressing cell line P2) were treated with increasing concentrations of Merb for 48 h, and their DNA was analyzed by PFGE. DMSO‐treated cells were used as controls (left panel). Quantification of Merb‐induced DNA fragments (> 1 Mb and 30–100 kb fractions; right panel) after normalization with total DNA (*n* = 3 independent experiments). (C) H1299 cells (parental and HMGA2 KO cells) were treated with 200 μm Merb for 48 h and their DNA was analyzed by PFGE. DMSO‐treated cells were used as controls (left panel). Quantification of Merb‐induced DNA fragments (> 1 Mb and 30–100 kb fractions; right panel) after normalization with total DNA (*n* = 3 independent experiments). Also, see Fig. S2A for an independent Merb titration experiment with H1299 cells. (A–C) See section 2.8 for statistical analysis.

**Figure 5:**
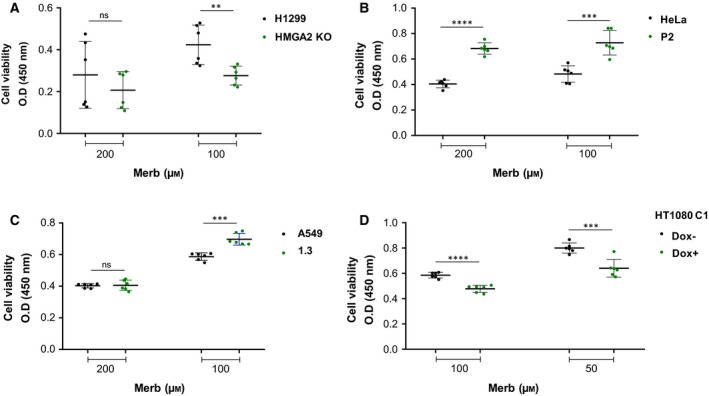
Effects of HMGA2 expression on cancer cell viability after Merb treatment *ex vivo*. Cell viability (CCK8) assay with (A) H1299 (parental and HMGA2 knockout cells), (B) HeLa (parental and HMGA2 expressing cell clone P2), (C) A549 (parental and HMGA2 expressing cell clone 1.3), and (D) HT1080 C1 (Dox−/Dox^+^ treated) cells after treatment with two different doses of Merb, as indicated, for 48 h (*n* = 2 independent experiments with 3 technical replicates for each experiment). (A–D) Unpaired two‐tailed *t*‐tests. Error bars, SD. **P < 0.01, ***P < 0.001, ****P < 0.0001, ns not significant.

Upon Merb treatment, PFGE revealed DNA damage profiles (30–100 kb and > 1 Mb) in all tested cancer cell models which are very similar to those induced by Etop (Fig. 4A–C and Fig. S2A). It appears, therefore, that excessive topological stress that results from the catalytic inhibition of TOP2 leads to DSBs which are similar to those generated by TOP2cc formation (McClendon and Osheroff, 2007; Tammaro *et al.*, 2013). Importantly, HMGA2 expression protected cells against Merb‐induced DSBs with a concomitant significant increase in cell survival (Fig. 5A–D).

**Figure 6:**
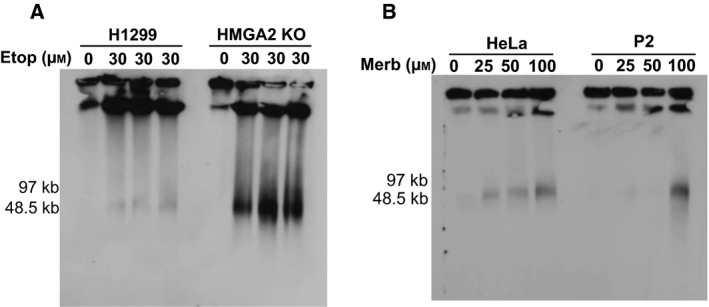
HMGA2 protects subtelomeric domains against TOP2‐induced DNA damage. (A) Southern blot with telomere‐specific probe for DNA from H1299 cells (parental and HMGA2 KO cells) analyzed by PFGE after treatment with 30 µm Etop for 24 h. See Fig. 1C for the corresponding EtBr stained PFGE image. (B) Southern blot with telomere‐specific probe of HeLa cells (parental and HMGA2 expressing cell clone P2) analyzed by PFGE after treatment with three doses of Merb for 48 h.

We have previously demonstrated that human subtelomeres are highly sensitive to inhibition of DNA replication triggered by the ribonucleotide reductase inhibitor HU, TOP1 poisoning by irinotecan (SN38), and the drug TMPyP4; the latter is thought to induce replication fork stalling by stabilizing so‐called G‐quadruplex DNA secondary structures. We proposed that subtelomeric regions were highly vulnerable to these genotoxic challenges due, at least in part, to strong DNA topological barriers located at human telomeres (Ahmed *et al.*, 2019). We therefore investigated whether targeting TOP2 with Etop and Merb also triggers DSBs at human subtelomeres. Southern blot analysis using telomeric DNA‐specific probes revealed similar fragment profiles that range from 30 to 100 kb (Fig. 6A–B). Based on our previous detailed analysis of the genomic DNA breakpoints leading to 30–100 kb subtelomeric DNA fragments (Ahmed *et al.*, 2019), these data strongly suggest that human subtelomeres are also cellular targets of TOP2 poisons and catalytic TOP2 inhibitors. Furthermore, these data corroborate our model that implicates a critical protective function for HMGA2 in regulating chromatin topological stress at these fragile genomic regions (Ahmed *et al.*, 2019).

Our recent studies showed that HMGA2 formed unique higher order complexes with scDNA *in vitro* and that within these ternary complexes, HMGA2 juxtaposes DNA segments into closer proximity to each other (Peter *et al.*, 2016; Zhao *et al.*, 2017). These results, in conjunction with the observed protective effect of HMGA2 against the catalytic inhibitor Merb, led us to investigate whether HMGA2 could catalytically activate TOP2A leading to more efficient supercoil relaxation. Because there is currently no reliable assay available that can directly determine DNA supercoil relaxation rates by TOPs inside cells, we employed standard TOP activity *in vitro* assays to address this question quantitatively.

**Figure 7:**
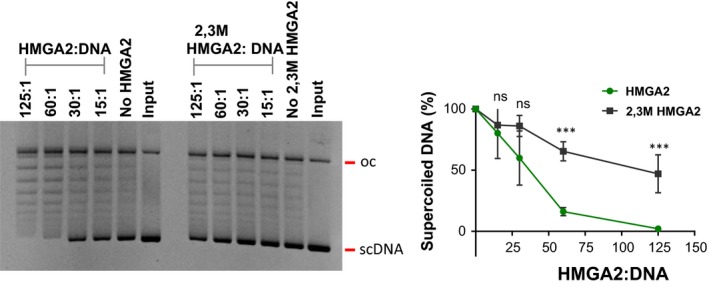
HMGA2 catalytically activates TOP2A *in vitro*. Supercoiled plasmid DNA was incubated with a trace amount of purified TOP2A, leading to only partially relaxed plasmid DNA during 30 min incubation [compare ‘input’ with ‘no (2,3M) HMGA2’ lanes]. The indicated HMGA2:DNA molar ratios were applied (left panel) and quantification of the HMGA2‐activated DNA relaxation by human TOP2A (by measuring only the amount of the remaining scDNA in a single band, as indicated at the right side of the left panel), revealed a significant stimulating effect by wild‐type, but not by the mutant HMGA2 (right panel; *n* = 3 independent experiments). Error bars, SD. Two‐way ANOVA followed by Sidak's multiple comparisons. ***P < 0.001. Quantification of scDNA band was done by imagej software. DNA was subjected to electrophoresis in 0.8% agarose gel and stained with ethidium bromide.

We found that during 30 min incubation with scDNA, HMGA2 greatly enhanced the relaxation activity of TOP2A, probably by promoting more productive TOP2A‐scDNA interactions at DNA crossings via DNA segment scrunching (Zechiedrich and Osheroff, 1990; Zhao *et al.*, 2017) (Fig. 7). This conclusion was strongly supported by results obtained with the 2,3M HMGA2 variant that harbored one instead of three functional AT‐hooks (Fig. 7). By employing this HMGA2 variant, we have previously shown that at least two functional AT‐hooks are required for efficient supercoil scrunching (Zhao *et al.*, 2017). Taken together, these data reveal that HMGA2 can attenuate the DNA damaging and cytotoxic effects of TOP2A poisons as well as of catalytic inhibitors in various cancer cells, and suggest that these protective effects may be triggered by DNA supercoil scrunching, and catalytic activation of TOP2A in the chromatin of cancer cells that express HMGA2.

## 4 Discussion

Topoisomerases 1/2 resolve topological ramifications in chromatin through controlled changes in the DNA linking number employing two distinct catalytic mechanisms. Interestingly, several chromatin/DNA architectural proteins have recently been implicated in TOP functions by stimulating DNA supercoil relaxation activity of TOPs (Guo *et al.*, 2018; Stros *et al.*, 2007; Stros *et al.*, 2009). In addition, several tumor‐associated proteins such as YB‐1 (Wu *et al.*, 2014), p53 (Gobert *et al.*, 1996) (Kwon *et al.*, 2000) and ARF (Karayan *et al.*, 2001) have been shown to enhance TOP activity either through direct protein–protein interactions or by enhancing TOP2 ATPase activity. Such findings could aid in our understanding of cellular chemoresistance that is often observed in cancer cells targeted with TOP poisons (Bansal *et al.*, 2017; Ganapathi and Ganapathi, 2013). In this context, our current study uncovered that HMGA2 is a crucial factor that can affect TOP2A function.

TOP2A plays a particularly important role during DNA replication in fast proliferating cells (Heck and Earnshaw, 1986; Hsiang *et al.*, 1988; Marinello *et al.*, 2018; Yan *et al.*, 2016), and while TOP2 poisons, such as Etop, are extensively utilized in clinical practice either as single agents or in combination therapy, the development of secondary tumors is still a major complication. Additionally, treatment efficacy depends on TOP2 expression and drug efflux pumps in cancer cells, thereby hampering their effectiveness as anticancer agents (Felix *et al.*, 2006; Nitiss, 2009; Ratain and Rowley, 1992; Vassetzky *et al.*, 1995).

Our *ex vivo* results presented in this study provide novel mechanistic insights into the regulation of TOP2‐mediated DNA damage and point at HMGA2 as a crucial factor in chemotherapeutic responses following exposure to TOP2 antagonists. Importantly, an extensive recent clinical study that included samples from more than 350 human *de novo* AML patients treated with TOP2 poisons alone or in combination with DNA synthesis inhibitors implicated HMGA2 expression in leukemic cells to poor clinical outcomes (Marquis *et al.*, 2018). These results were further validated in more than 250 patients, thus highlighting its clinical significance (Marquis *et al.*, 2018). Importantly, these clinical data directly correlate with our *ex vivo* findings clearly revealing a protective role for HMGA2 against TOP2A targeting drugs and, taken together, illustrates their importance for clinical strategies in particular for HMGA2‐positive AML patients.

With more than 60% of AML patients succumbing to leukemia‐related issues, and with high HMGA2 expression correlating to poor survival in both the experimental and validation groups (Marquis *et al.*, 2018), we postulate here that resistance to treatment is, at least in part, due to both HMGA2's ability to catalytically activate TOP2A and to serve as replication fork chaperone during induced replication stress using DNA synthesis inhibitors; the latter role for HMGA2 has been demonstrated previously in other cancer cell models (Ahmed *et al.*, 2019; Yu *et al.*, 2014). More than 70% of AML patients with elevated HMGA2 expression levels experienced relapse after complete remission. Bone marrow‐derived mononuclear cells that were tested positive for high HMGA2 expression (Marquis *et al.*, 2018) comprise of a myriad of cells at different stages of maturation, with hematopoietic stem (HPS) cells playing a central role in the development of AML (Corces *et al.*, 2017; Cuende *et al.*, 2012; Sanchez‐Aguilera and Mendez‐Ferrer, 2017). Importantly, HMGA2 is found to be expressed in human HPS and progenitor cells and serves an important function in proliferation and maintenance of cellular multipotency (Copley *et al.*, 2013; Kumar *et al.*, 2019). A protective function against TOP2‐targeting drugs in transformed HPS cells, as exemplified in our current study with various other cancer cell models, as well as the HMGA2‐mediated protection against DNA synthesis inhibitors such as HU (Ahmed *et al.*, 2019; Yu *et al.*, 2014), could therefore be critical factors for the increased occurrence of AML relapse.

**Figure 8:**
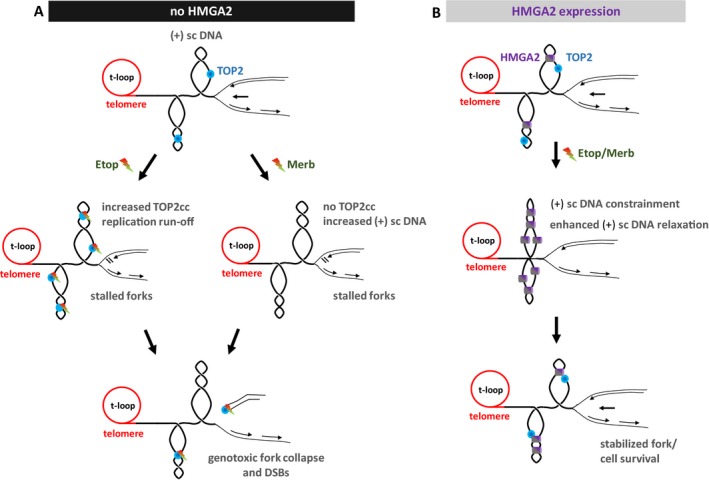
Model of the role of HMGA2 in protection of replication forks at subtelomeres against TOP2 targeting drugs. (A) In the absence of HMGA2, TOP2 inhibition by either Merb or Etop generates high levels of (+) supercoiled (sc) DNA ahead of the replication fork or an increase in TOP2cc formation, leading to frequent fork collapse and replication runoff events, respectively. (B) HMGA2 constrains (+) sc DNA and activates TOP2A within transient ternary complexes to relax (+) sc DNA, thus preventing fork collapse/DSBs.

We identified that fragile sites such as subtelomeres (Sfeir *et al.*, 2009) are susceptible to DNA topological challenges generated by TOP2 inhibition and found that subtelomere instability is also influenced by HMGA2, hence corroborating our previous study which investigated TOP1 poisons and HU (Ahmed *et al.*, 2019). Interestingly, the recently discovered bacterial chromosome factor GapR contributes to genome stability especially during fast replication cycles through DNA supercoil constrainment and activation of gyrase (a bacterial TOP2) within transient DNA–protein ternary complexes (Guo *et al.*, 2018). We propose here a very similar role for HMGA2 and TOP2A which protects subtelomeric and other human genomic regions against induced, unscheduled fork stalling/collapse and chromosomal breakage. In cells lacking HMGA2, treatment with Etop will enhance the occurrence of TOP2cc formation downstream of replication forks and results in frequent replication runoff events and genotoxic DNA damage that releases telomeric DNA fragments (Fig. 8A). This model is supported by the results from a recent study that demonstrated human TOP2 facilitates telomere replication and that TOP2 poisoning leads to excised telomeric DNA circles as a result of replication fork runoff/collapse (Zhang *et al.*, 2017). Treatment with the catalytic inhibitor Merb, on the other hand, will primarily lead to excess of (+) supercoiling downstream of progressing forks, which, in turn, promotes fork stalling and collapse into genotoxic DSBs following nucleolytic attack (Fig. 8A). Interestingly, a recent study showed that inhibition of the catalytic activity of TOP2A leads to telomere fragility during mitosis, most likely through the formation of unresolved catenated DNA between sister chromatids (d'Alcontres *et al.*, 2014). In cancer cells expressing HMGA2, we propose that the protein mitigates the effects of both TOP2 targeting drugs by a combination of DNA supercoil constrainment and TOP2A catalytic activation, hence acting as a DNA supercoil ‘sink’ (Fig. 8B).

Our *in vitro* results obtained with the HMGA2 variant that carries substitutions in AT‐hooks 2 and 3 imply that the supercoil constrainment could directly lead to the catalytic activation of TOP2. However, this does not exclude that this catalytic activation function may also be mediated by direct HMGA2‐TOP2 physical interactions, as identified through a HMGA2 interactome study using mouse cells (Singh *et al.*, 2015). Such protein–protein interaction would aid TOP2 to more efficiently recognize and associate with relevant regions in supercoiled substrates such as AT‐rich nuclear matrix attachment sites within a highly compacted and entangled chromatin. This scenario is also supported by studies that demonstrated chromatin colocalization between HMGA proteins and TOP2 (Martelli *et al.*, 1998; Reeves, 2010; Saitoh and Laemmli, 1994).

Our study revealed that the catalytic inhibitor Merb that does not lead to the formation of TOP2cc generates DNA damage profiles similar to those observed with TOP2 poisons, implying that excess topological stress alone due to an overall reduced intracellular TOP2 activity can cause genotoxic DNA damage. Based on the very similar DNA damage profiles that we observed after inhibiting DNA synthesis by HU (Ahmed *et al.*, 2019; Yu *et al.*, 2014), we think that the collapse of stalled replication forks triggered by unresolved DNA topological stress could be a more frequent cause for the genotoxic effects of these different and clinically relevant drugs. This contests the widely accepted notion that the formation of protein–DNA adducts (TOP2cc) on the parental strands and subsequent collision with replisomes is solely responsible for fork collapse and a major contributor to DSBs and genome instability (Larsen *et al.*, 2003; Nadas and Sun, 2006).

Our current study investigated a role of HMGA2 in the induction of DNA lesions during the course of genotoxic drug treatment. However, HMGA2 has recently also been implicated in DNA damage repair signaling pathways, thus hinting at important functions in genome stability that act downstream of the formation of DNA lesions (Hombach‐Klonisch *et al.*, 2019; Natarajan *et al.*, 2013; Palmieri *et al.*, 2011). In the context of AML, it will be interesting to investigate in the future whether these functions cooperate in leukemic cells to counteract chemotherapy. Furthermore, it will be interesting to determine whether treatment with the TOP1 poison irinotecan/SN38 instead of the TOP2 poison Etop leads to improved outcomes in AML patients expressing low‐to‐moderate HMGA2 levels in leukemic cells, as implied by the results of our previous work (Ahmed *et al.*, 2019).

## 5 Conclusions

In conclusion, the attenuation of genotoxic DSBs during Etop or Merb treatment in the presence of HMGA2 highlights its role in regulating DNA/chromatin topological stress genomewide and especially at fragile subtelomeric regions. The latter are prone to replication fork stalling due to their heterochromatic nature, terminal position, and the potential formation of DNA topological barriers in form of T‐loops (Cubiles *et al.*, 2018; Martinez and Blasco, 2015). This suggests a novel mechanism of chemoresistance toward combination therapies involving TOP2 poisons/inhibitors and strongly argues for HMGA2 expression profiling to aid therapy decision making, in particular in AML patients.

## Conflict of interest

The authors declare no conflict of interest.

## Author contributions

SMA and PD designed the study. SMA performed the experiments and analyzed the data. SMA and PD wrote the manuscript. PD supervised and obtained funding for the study.

## Supporting information


**Fig. S1** **.** HMGA2 protects against DNA damage induced by Etop. (A) HeLa cells were treated with increasing concentrations of Etop for 24 h and their DNA was analyzed by PFGE. (B) H1299 cells (parental and HMGA2 KO cells) were treated with increasing concentrations of Etop for 24 h and their DNA was analyzed by PFGE. (C) HT1080 C1 cells with Dox‐regulated HMGA2 expression were treated with 10 μm Etop for 24 h and their DNA was analyzed by PFGE (left panel). Quantification of Etop‐induced DNA fragments (> 1 Mb and 30–100 kb fractions) (right panel) after normalization with total DNA (*n* = 3 independent experiments). See section 2.8 for statistical analysis.

**Fig. S2** **.** HMGA2 protects against DNA damage induced by Merb. (A) H1299 cells (parental and HMGA2 KO cells) treated with decreasing concentrations of Merb for 48 h and their DNA was analyzed by PFGE.

## Data Availability

The datasets for this study including raw data files for all figures along with supplementary material can be found on Mendeley dataset at: https://data.mendeley.com/datasets/fnztgsz6zh/draft?a=76385a4f-81bd-4fa9-b9fe-4cbe04ff8042
