# Supplementary figures and images for: Oncofetal HMGA2 attenuates genotoxic damage induced by topoisomerase II target compounds through the regulation of local DNA topology
Source: Mol Oncol. 2019 Aug 31;13(10):2062–78. doi: 10.1002/1878-0261.12541 (PMC6763970; doi:10.1002/1878-0261.12541)

**A**

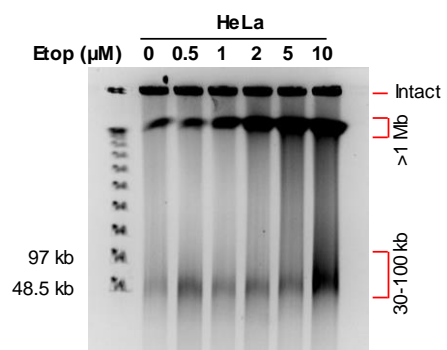

**B**

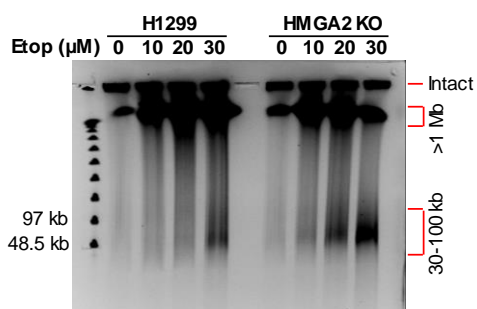

**C**

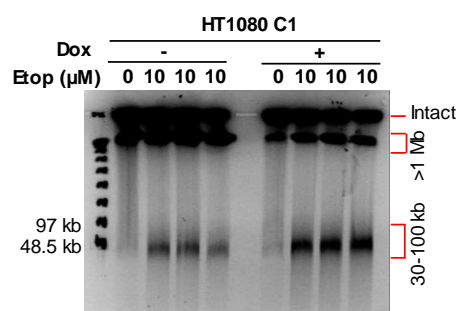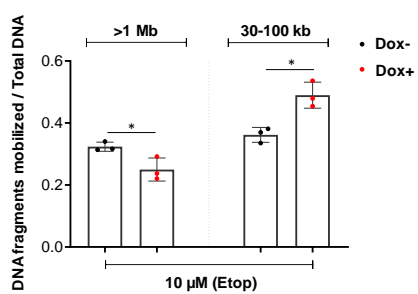

Supplement: Supplementary file 1 — Fig. S1 . HMGA2 protects against DNA damage induced by Etop. (A) HeLa cells were treated with increasing concentrations of Etop for 24 h and their DNA was analyzed by PFGE. (B) H1299 cells (parental and HMGA2 KO cells) were treated with increasing concentrations of Etop for 24 h and their DNA was analyzed by PFGE. (C) HT1080 C1 cells with Dox‐regulated HMGA2 expression were treated with 10 μm Etop for 24 h and their DNA was analyzed by PFGE (left panel). Quantification of Etop‐induced DNA fragments (> 1 Mb and 30–100 kb fractions) (right panel) after normalization with total DNA (n = 3 independent experiments). See section 2.8 for statistical analysis. [file MOL2-13-2062-s001.pdf]

A

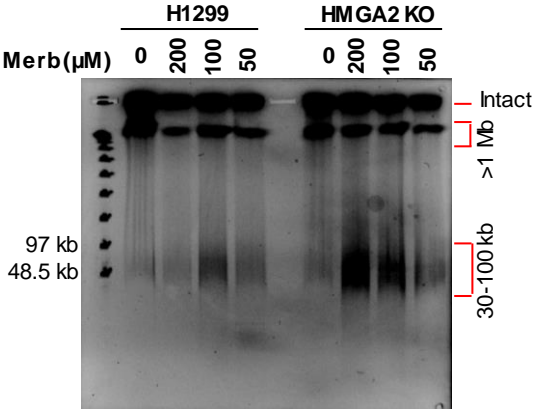

Supplement: Supplementary file 2 — Fig. S2 . HMGA2 protects against DNA damage induced by Merb. (A) H1299 cells (parental and HMGA2 KO cells) treated with decreasing concentrations of Merb for 48 h and their DNA was analyzed by PFGE. [file MOL2-13-2062-s002.pdf]
